# Ontological interpretation of biomedical database content

**DOI:** 10.1186/s13326-017-0127-z

**Published:** 2017-06-26

**Authors:** Filipe Santana da Silva, Ludger Jansen, Fred Freitas, Stefan Schulz

**Affiliations:** 1 Centro de Informática, Universidade Federal de Pernambuco, Av. Jornalista Anibal Fernandes, 50.740-560, Recife, Brazil; 2 Núcleo de Telessaúde, Universidade Federal de Pernambuco, Av. Prof. Moraes Rego, 50670-420, Recife, Brazil; 3 Institut für Philosophie, Universität Rostock, D-18051, Rostock, Germany; 4 Institute for Medical Informatics, Statistics and Documentation, Medical University of Graz, Auenbruggerplatz 2/V, Graz, 8036 Austria

**Keywords:** Ontology, Interpretation, Biological database, OWL, Data semantics

## Abstract

**Background:**

Biological databases store data about laboratory experiments, together with semantic annotations, in order to support data aggregation and retrieval. The exact meaning of such annotations in the context of a database record is often ambiguous. We address this problem by grounding implicit and explicit database content in a formal-ontological framework.

**Methods:**

By using a typical extract from the databases UniProt and Ensembl, annotated with content from GO, PR, ChEBI and NCBI Taxonomy, we created four ontological models (in OWL), which generate explicit, distinct interpretations under the BioTopLite2 (BTL2) upper-level ontology. The first three models interpret database entries as individuals (IND), defined classes (SUBC), and classes with dispositions (DISP), respectively; the fourth model (HYBR) is a combination of SUBC and DISP. For the evaluation of these four models, we consider (i) database content retrieval, using ontologies as query vocabulary; (ii) information completeness; and, (iii) DL complexity and decidability. The models were tested under these criteria against four competency questions (CQs).

**Results:**

IND does not raise any ontological claim, besides asserting the existence of sample individuals and relations among them. Modelling patterns have to be created for each type of annotation referent. SUBC is interpreted regarding maximally fine-grained defined subclasses under the classes referred to by the data. DISP attempts to extract truly ontological statements from the database records, claiming the existence of dispositions. HYBR is a hybrid of SUBC and DISP and is more parsimonious regarding expressiveness and query answering complexity. For each of the four models, the four CQs were submitted as DL queries. This shows the ability to retrieve individuals with IND, and classes in SUBC and HYBR. DISP does not retrieve anything because the axioms with disposition are embedded in General Class Inclusion (GCI) statements.

**Conclusion:**

Ambiguity of biological database content is addressed by a method that identifies implicit knowledge behind semantic annotations in biological databases and grounds it in an expressive upper-level ontology. The result is a seamless representation of database structure, content and annotations as OWL models.

**Electronic supplementary material:**

The online version of this article (doi:10.1186/s13326-017-0127-z) contains supplementary material, which is available to authorized users.

## Background

Biological databases store data about summarized results from laboratory experiments. Apart from numeric and unstructured text entries, they usually include semantic annotations, characterized by identifiers from domain ontologies, to enhance database entries with standardised meaning. For instance, database records from the Unified Protein Resource (UniProt) [1] are annotated with terms taken from the Protein Ontology (PRO) [2] and the Gene Ontology (GO) [3]. It is mainly via their use as annotation vocabularies that bio-ontologies have become important resources for the management of biomedical research data.

As much as these domain ontologies, in isolation, obey formal principles and good practice guidelines [4, 5], as little the meaning of the annotations themselves has been formalized so far. The exact interpretation of what it means when, e.g., in a UniProt record the protein PRO:*Methionine synthase* is linked to the biological process GO:*Methylation*, is left to the user, mainly due to limited representation of UniProt Core [6]. UniProt Core includes the description on database fields related to each other, but without formalization and links to GO (for example). This can constitute a source of misunderstanding and hamper correct data interpretation, leading to doubtful or wrong conclusions.

Although the meaning of semantic annotations in database records may seem trivial for domain experts, human interpretation of large numbers of records is tedious and time-consuming. Laukens and colleagues [7], among others, have highlighted the difficulty of interpreting database content in the context of proteomics. The reason for this is that there is still a divide between biological databases and the semantic technologies developed for biomedical ontologies. Scattered data need to be integrated into a coherent picture, which is complicated by ambiguity and lack of interoperability.

On the one hand, there are rich and well-curated databases with highly structured tabular content but limited ontological explicitness. Like most content of tabular data structures, these databases require implicit background assumptions for their correct interpretation.

Imagine, for example, a database table with three fields *Protein*, *Organism* and *Phenotype*, filled with the symbols *Prot*_1_, *Org*_1_, and *Phen*_1_. Such a table is open to multiple interpretations, among which only one is the intended one, viz. that organisms of the type *Org*_1_ in which protein *Prot*_1_ is dysfunctional are at risk to develop the pathological phenotype *Phen*_1_. This interpretation is not formally described anywhere, because it is assumed that database curators and users would not succumb to erroneous interpretations, such as that all proteins of *Prot*_1_ are included in at least one organism of type *Org*_1_, or that organisms of type *Org*_1_ have as part at least one protein of the type *Prot*_1_ and exhibit specifically at least a *Phen*_1_. Therefore, a formal description would be fundamental for the correct interpretation of the database content in other contexts.

On the other hand, there is an increasing number of biomedical ontologies in which logic-based axioms provide precise descriptions, which indeed enable formal reasoning. Such axioms are expressed in Description Logics (DL) [8] using the Web Ontology Language OWL2 [9]. DL queries can be answered based on satisfiability testing and class subsumption. For instance, such queries enable to retrieve Parkinson’s disease in a query when searching for diseases that affect the extra-pyramidal system, if Parkinson’s disease has been formally characterised as a disorder located in the basal ganglia of the brain, and the latter as part of the extra-pyramidal system.

This division between database content and structure on the one hand (with its implicit meaning) and ontology content on the other hand (with its explicit meaning) is, currently, an obstacle towards querying both together. Given this picture, several questions arise:

i. How can the implicit knowledge about entities and relationships described in the *structure* of a biological database be represented?
ii. How can the *content* of databases be interpreted, i.e., which domain entities are represented by the data elements and their connections?
iii. Are structure and content of biological databases of ontological nature?
iv. If this is the case, how can they be translated into axioms or assertions in a commonly used ontology language, and which representational patterns might be considered?
v. Once database structure and content are expressed by formal-ontological means, how can existing bio-ontologies be plugged into this structure?
vi. Given a seamless integration among these components, are there benefits for content retrieval, regarding correctness, completeness, and user-friendliness?
vi. Is such a system capable to accommodate large amounts of data in biological databases, also considering the size of a domain ontology?

Addressing questions i-iv, we hypothesise that there are feasible ways to express implicit and explicit database content by formal-ontological means and combine this content with pre-existing domain ontologies.

Regarding question v, previous work has shown how content of tables in scientific publications can be interpreted on formal grounds [10]. Question vi has been addressed in [11], which introduced the reasoning capabilities of querying highly axiomatised bio-ontologies. Question vii needs to be addressed after answering questions i-iv, but is beyond the scope of the present paper.

We will demonstrate how entities referenced by a typical extract from a biomedical database can be interpreted under several ontological viewpoints, *viz.* regarding the introduction of individuals (IND), the addition of new axioms to existing classes (DISP) and the introduction of additional defined classes (SUBC and HYBR). The resulting OWL models are, then, tested under three aspects:

i. Database content retrieval: classes or individuals are retrieved by means of DL queries;
ii. Information completeness: is the interpretation generated able to answer user queries?
iii. DL complexity and decidability: in order to solve DL queries, there should be theoretical guarantees that the machine performs under a reasonable cost and finite time (complexity) and always finishes its task (decidability).

## Methods

This section describes the ontology engineering principles we subscribed to, as well as the data we gathered to exemplify our approach.

### Engineering principles

Firstly, we believe that ontology structure and content should be driven by the underlying reality, rather than by specific application needs. We subscribe to the principles of the OBO Foundry [4], and emphasise the use of a principled upper-level ontology, here BioTopLite2 (BTL2) [12], which offers a set of high-level classes, together with constraining axioms, using a small number of core relations. Classes like *Organism*, *‘Mono molecular entity’*, and *‘Body part’* facilitate the alignment with other ontologies like GO, PRO, SNOMED CT and ChEBI. BTL2 can also be aligned with most of BFO [13] and the OBO Relation Ontology [14]. BTL2 regards all instances of its classes as implicitly time-indexed, thus solving the ambiguity problem of using binary relations for the cases where BFO2 [13] requires ternary ones, which are not expressible in OWL [15].

The fundamental role of Description Logics (DLs) [8] is justified by the widespread use of the Web Ontology Language OWL2 [9], supported by popular editors and classifiers [16]. We use OWL-DL, which corresponds to the language specification *SROIQ* [17], and which combines expressiveness with complete and finite reasoning power. OWL2 supports classes, binary relations (object properties), and individuals, together with related axioms and assertions, for which we will use the OWL2 Manchester Syntax [18]. Important for DL is the distinction between ABox and TBox. The TBox contains “terminological" class-level axioms, i.e. the ontological content proper, whereas the ABox contains contingent “assertions" about individuals.

### Dispositions

Real world entities are often described in terms of dispositions, i.e., tendencies of something to act in a certain manner under given circumstances resulting from natural constitution, nature, quality, or orderly arrangement. Saying that all animals are organisms is a universal statement; stating that all humans are able to develop diabetes mellitus type 2 is a dispositional statement. Several works [12, 19–21] have suggested to include dispositions in biomedical ontologies; e.g., the disposition to pump blood is present in all healthy organs of the type *Heart*.

Large parts of biomedical database content seem to be dispositional: In biochemistry, a statement that a protein *A* participates in a process *B* does probably not mean that all instances of *A* constantly participate in a process of type *B*, but rather that all instances of A have the disposition to participate in such a process. Biomedical observations yield statistical results, which indicate that participants of an experiment are ascribed to certain capabilities (e.g. to participate in *B* under certain experimental conditions) [19, 22].

### Information content entities

Finally, database content as such needs ontological scrutiny, as highlighted in [7]. Database content is ontologically best characterised as information content. This requires a strict distinction between (i) the database content proper and (ii) the entities in the world referenced by the former. As well as the data in clinical documents, biomedical database content is connected by a specific relation (often named “represents”, “isAbout”, or “denotes”) with biomedical entities. Such information content entities do not necessarily denote particulars (i.e., instances) in the domain described. A “myocardial infarction” record entry about a patient recently admitted to the emergency room may have the attribute “probable”, even if the patient does (in fact) not have any heart problem. Similarly, a database entry on, e.g., the relation between protein *P*_*k*_ and phenotype *T*_*i*_ in an organism *O*_*m*_ may be affected by experimentation, reporting, or curation errors.

### Running example

For the analysis reported in this paper, we selected a typical biological database example (cf. Table 1), generated by joining data from UniProt [1] and Ensembl [23] by standard database querying (Additional file 1). This was performed in order to retrieve all related records to the metabolism of homocysteine and other sulphurated amino acids, like methionine and cysteine (see [24] for more information regarding homocysteine metabolic pathway).

**Table 1:** Typical data record from the joined databases Uniprot and Ensembl. The abstraction introduces the symbols of the example ontologies

Field	Source	Content	Abstraction
Protein (PR)	UniProt	Cystathionine gamma-lyase	*Prot*_1_
Organism (NCBI Taxonomy)	NCBI Taxonomy via UniProt	*Rattus norvegicus* (Rat)	*Org*_1_
Processes (not distinguishing between ‘*Biological process*’ and ‘*Molecular function*’ in Gene Ontology (GO))	GO via UniProt	hydrogen sulfide biosynthetic process; negative regulation of apoptotic signaling pathway; positive regulation of I-kappaB kinase/NF-kappaB signaling; protein homotetramerization; protein sulfhydration	*BProc*_1_, *BProc*_2_, …, *BProc*_*k*_
Cell components (GO_cc)	GO via UniProt	cytoplasm; nucleus; extracellular vesicular exosome;	*CComp*_1_, *CComp*_2_, …, *CComp*_*x*_
Small molecules (ChEBI)	ChEBI	Homocysteine	*Mol*_1_, *Mol*_2_, …, *Mol*_*y*_
Phenotypes	Ensembl	Amino acid metabolism errors; cataract; Gamma-cystathionase deficiency	*Phen*_1_, *Phen*_2_, …, *Phen*_*z*_

From UniProt (release 2015_01), we retrieved 21,868 records, and (exactly) 1000 from Ensembl (release 78). All sample data were retrieved on January 22^nd^, 2015. Data from the NCBI Taxonomy (2015AA) were incorporated at the end of the retrieval process, adding the taxonomy identifiers of the organisms from which data are recorded in UniProt and Ensembl.

Using the ontology editor Protégé v.5, supported by the DL classifier HermiT [16] v.1.8.3, we created four OWL2 models, each of which followed a different strategy. They were created according to the data organisation presented in Table 1, based on a sample record (Table 2). Terms for individuals were created according to the same organization, but identified by a bold lower-case letter and a random number, like „**p**_1001_“ or „**m**_2001_“ as terms for an individual protein and molecule (respectively).

**Table 2:** Schematic view over UniProt, NCBI Taxonomy and Ensembl data

Protein	Organism	Bio Process	Cell component	Molecule	Phenotype
*Prot*_1_	*Org*_1_	*BProc*_1_;	*CComp*_1_;	*Mol*_1_;	*Phen*_1_;
		*Bproc*_2_;	*CComp*_2_;	*Mol*_2_;	*Phen*_2_;
		*Bproc*_3_	*CComp*_3_	*Mol*_3_	*Phen*_3_

The four OWL models uniformly represent all information entities (database content) as individuals. The models differ, however, in the way how referents of this information are interpreted, viz. (i) as individuals (Additional file 2), (ii) as fully defined subclasses (Additional files 3 and 4) (iii) as disposition (Additional file 5) classes.

In the following, names of individuals are picked out in **bold face** with lower case initials, in contrast to class names in *italics* with leading upper case character. Symbols that include white spaces are enclosed in single quotes, e.g., ‘**has part**’.

In order to test the fitness of these models, four competency questions (CQs) were formulated in natural language and then reformulated as DL queries (cf. Table 3) in order to emulate typical query operations over ontologies and databases, performed by biomedical researchers. Q1 aims at retrieving biological processes in which certain proteins participate; Q2 retrieves the cellular component(s) a given organism includes, together with the proteins found in them. Q3 retrieves proteins recorded as participant of biological processes in a given organism. Finally, Q4 retrieves organisms able to exhibit a specific phenotype.

**Table 3:** Queries translated into DL queries

Q1 – Which biological processes have proteins of the kind *Prot*_1_ as participant?
*‘Biological process’* and (‘**has participant**’ some *Prot*_1_)
Q2 – In which cellular locations is *Prot*_1_ active in organisms of the type *Org*_1_?
‘*Cellular component*’ and (‘**is included in**’ some *Org*_1_) and
(**includes** some *Prot*_*i*_)
Q3 – Which proteins are involved in processes of the type *BProc *_1_ in organisms of the type *Org*_1_?
*Protein* and (‘**is participant in**’ some *BProc*_1_) and (‘**is included in**’ some *Org*_1_)
Q4 – Which organisms are able to exhibit a specific phenotype *Phen*_1_?
*Organism* and (‘**is bearer of**’ some (*Disposition* and (‘**has realization**’ only *Phen*_1_)))

## Results

Table 1 represents the typical structure of the data analyzed in this work. It is categorized and organized by the following structure:

- one protein term (e.g., *CBS)*;
- one taxon term (e.g., *Rattus norvegicus*);
- one to many terms for GO biological processes or GO molecular function (e.g., *‘Blood vessel remodelling’*);
- one to many terms for GO cellular components (e.g., *Cytoplasm*);
- zero to many terms for phenotypes (e.g., *‘Endocrine pancreas increased size’*);
- one to many terms for small molecules (e.g., *Homocysteine*)

This structure was imported from UniProt and expanded with mappings to Ensembl via identifiers. Following [25], we treat terms from GO ‘*Molecular function*’ as referring to processes. This is supported by the fact that the latter ones are named “activities” in GO; and heuristically, by the fact that in experiments molecular functions are always discovered through their realizations, i.e., through the observation of processes or their results.

Even if all terms from the database are understood, there are still numerous open questions regarding the precise meaning of such a database record. We fill this gap by eliciting the necessary implicit knowledge from a domain expert familiar with the process of database population, performing an in-depth ontological analysis in the line of Gangemi et al. [26]. This analysis begins with the formal categorization of relations and basic classes, under a suitable upper-level ontology. This was done by manually aligning the top-level classes of the domain ontologies GO, ChEBI and PR under the top-level ontology BTL2 [12].

Once the entities are categorised, the following questions need to be answered:

- How are the structural elements of a database (i.e. tables, fields) related to each other? Which knowledge is missing that is required for correctly understanding these relations?
- Which expressiveness is required to axiomatise the content in a logic-based language in an appropriate way to represent all implicit and explicit content?
- Which additional entities need to be included into the ontology (e.g., *Dysfunctionality* and *Disposition* in the above example)?
- Which compromises and simplifications may be needed? Which propositions are categorical, which ones are dispositional? [19] Do we have to include ABox entities (individuals)?

When it comes to an ontology-based representation of database content (as exemplified in Table 1), we face three interpretation challenges: (i) the data points and column headers, (ii) the relation between the data points and the column headers, and (iii) the relations among the columns.

Task (i) is facilitated by the fact that many of the content terms are already represented in biomedical ontologies like GO. Besides, the natural language terms used as field labels can easily be aligned to content from other ontologies. In our case, most field labels could be aligned with BTL2.

Task (ii) will normally be accounted for by the subclass or instantiation relation: the content terms denote classes or instances of the class denoted by the field label. E.g., *‘Cystathionine gamma-lyase’* subClassOf *Protein*, *‘Rattus norvegicus’* subclassOf *Organism*, etc.

Task (iii) requires reference to the implicit knowledge a scientist is likely to have. For example, a UniProt record that points to *Methylation*, *Bos taurus* and *‘Methionine synthase’* expresses that in a given experiment with cattle tissue an instance of *‘Methionine synthase’* was observed to participate in a methylation process.

In the following, we investigate four different approaches for representing the meaning of the content and structure of biological databases:

1. Representation as sample individuals (IND);
2. Representation as defined maximally fine-grained subclasses, seeing as referents of the information entities in the database (SUBC);
3. Representation with dispositional properties (DISP);
4. Hybrid representation with subclasses and dispositions (HYBR).

Our sample ontologies include one *Protein* class (*Prot*_1_), one *Organism* class (*Org*_1_), and three subclasses of each of *‘Cell Component’* (*CComp* $_{1_{,\ldots,} 3}$), *‘Biological Process’* (*BProc* $_{1_{,\ldots,}3}$), *‘Small Molecule’* (*Mol* $_{1_{,\ldots,}3}$), and *Phenotype* (*Phen* $_{1_{,\ldots,}3}$), respectively (Table 2).

### Representation as individuals (IND)

The first representation is motivated by the fact that a database entry is about a concrete experiment, in which individual entities in space and time are described, e.g., a piece of biological material, a certain amount of molecules, the phenotype of an individual rat, etc. This view is agnostic with respect to whether the observed phenomena are manifestations of natural laws or not.

In this perspective, our sample data report that individual protein molecules **p**_**1001**_, **p**_**1002**_, …of the type *Prot*_1_ exist in some particular cell components **cc**_**1001**_, **cc**_**2001**_, …of the types *CComp* $_{1_{,\ldots,}n}$ of some organisms **o**_**1001**_, **o**_**1002**_, …of the type *Org*_1_. Biomolecular process individuals **bp**_**1001**_, **bp**_**2001**_, …that are members of the classes *BProc* $_{1_{,\ldots,}m}$ include molecules **m**_**1001**_, **m**_**2001**_, …of the type *Mol* $_{1_{,\ldots,}k}$ (specific to *Org*_1_). Finally, the dysfunctions of the proteins **p**_**1001**_, **p**_**1002**_, …cause the organisms **o**_**1001**_, **o**_**1002**_, …to display one or more phenotypes **ph**_**1001**_, **ph**_**2001**_, …of the type *Phen* $_{1_{,\ldots,}n}$ (Table 2).

We are aware that only collections of molecules (and never single molecules) and activities thereof are observed [22]. However, assuming that the observation of the behaviour of collective individuals allows us to deduce what happens at the level of individuals (as done when describing chemical reactions or biochemical pathways with symbols denoting single molecular entities), we here populate the ABox with single, non-collective, sample entities and the relations among them. Index numbers are aligned arbitrarily.

In the following we describe our interpretation approach. For instance, individual protein molecules in individual organisms are active in processes, e.g., within cell components, like:

**p**_**1001**_ **‘is included in’ cc**_**1001**_

**cc**_**1001**_ **‘is included in’ o**_**1001**_

We also introduce instances for protein molecules that participate in process instances within an organism:

**p**_**1004**_ **‘is participant in’ bp**_**1001**_

**p**_**1004**_ **‘is included in’ o**_**1004**_

Protein molecules participate, within a particular organism, in process instances (e.g., **bp**_**1001**_) that synthesise specific molecules (e.g., **m**_**1001**_):

**p**_**1010**_ **‘is participant in’ bp**_**1001**_

**bp**_**1001**_ **‘has participant’ m**_**1001**_

**p**_**1010**_ **‘is included in’ o**_**1010**_

Whenever the database fields for processes, molecules, or cell components have more than one entry, the database, unfortunately, leaves open which processes involve which molecules and where they are located. Ideally, this information might be retrieved from other sources. Otherwise, a relation between an individual processes and molecules participating in them can be expressed by referring to an appropriate process individual **bp**_**1001**_ and an appropriate individual molecule **m**_**1001**_. An analogous strategy is possible to express the participation of cell components in processes.

**bp**_**1001**_ **includes** **m**_**1001**_

There are organisms with specific phenotypes, in which there is a protein of a certain type, which is however dysfunctional. Dysfunctionalities can be represented as qualities, here also expressed as the individual **d**_**1001**_.

**p**_**1013**_ **‘is included in’ o**_**1013**_

**o**_**1013**_ **‘includes’ ph**_**1001**_

**p**_**1013**_ **‘is bearer of’ d**_**001**_

For these data to be interpreted in a DL context, ABox entities (in this scenario) are to be understood as arbitrary individuals that participate in a specific experiment. For the sake of simplicity, for each assertion that can be derived from the database, new terms for individuals are created.

Another simplifying assumption of this approach is that all database terms are non-empty, i.e., they actually refer to some existing entity. Each information-content individual in the database needs to represent an existing individual involved in the experiment. This is, of course, problematic if the data is wrong due to curation errors, or if the biological processes recorded did not really happen.

### Representation as multiple subclasses (SUBC)

**Fig. 1:**
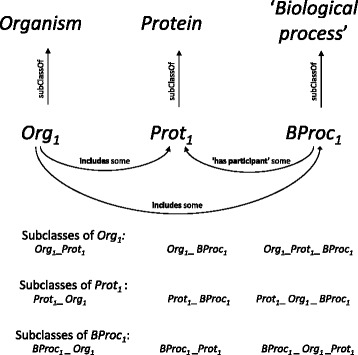
Example of subclass creation and relations enabled to be used in class definitions

The second approach interprets database terms as referring to maximally fine-grained defined classes. The naming of these new subclasses follows strict naming criteria as exemplified below. This is important for extracting the original class names from the subclass names, because only the former ones are interesting for querying. For instance, the database represents a protein class *Prot*_1_ that is connected with an organism class *Org*_1_ and a bioprocess class *BProc*_1_. Accordingly, we create the classes *Prot*_1_ *_in_Org*_1_ *_in_BProc*_1_, *Org*_1_ *_with_Prot*_1_ *_and_BProc*_1_, and *BProc*_1_ *_in_Org*_1_ *_with_Prot*_1_ with appropriate full definitions (Fig. 1).

We leave open whether these defined classes are empty. In a way, defined classes are nothing more than logical artefacts. For this reason, the creation of such defined OWL classes has a modest ontological engagement. Nevertheless, these defined classes can serve as the referents of the data instances [27].

In order to fully incorporate the idea that database entries are individuals that refer to classes by means of annotations, we create the following description logic formula for each database entity:

**databaseEntry**_***x***_ type **represents** only

(*DefinedClass*_1_ or *DefinedClass*_2_ or …or *DefinedClass*_*N*_)

Bearing this representation in mind, querying can be limited to the expression in parentheses, which brings two advantages, viz. that neither individuals and nor value restrictions would impact the performance of the reasoner.

In the following, the modelling patterns are given for proteins, organisms, small molecules, biological processes and phenotypes. Here, the index variable *i* denotes a record, in which field (*e.g.*, for protein) is filled exactly once; hence the notation *Prot* $_{i_{1}}$. Accordingly, the notation for organisms is *Org* $_{i_{1}}$, because there is exactly one organism type referred to by a record. The other fields may be multiply filled; therefore the notation is, e.g., *BProc* $_{_{1}}$, *BProc* $_{_{2}}$, …, *BProc* $_{_{m}}$.

**Proteins**: We introduce classes for dysfunctional proteins as well as for organism-specific proteins and their combination:

*Prot* $_{i_{1}}$ *_Dysf* equivalentTo *Prot* $_{i_{1}}$ and

‘**is bearer of**’ some *Dysfunctional*

*Prot* $_{i_{1}}$ *_in_Org* $_{i_{1}}$ equivalentTo *Prot* $_{i_{1}}$ *and*

‘**is part of**’ some *Org* $_{i_{1}}$

*Prot* $_{i_{1}}$ *_Dysf_in_Org* $_{i_{1}}$ equivalentTo

*Prot* $_{i_{1}}$ *_Dysf* and *Prot* $_{i_{1}}$ *_in_Org* $_{i_{1}}$

Specifically, subclasses are created to represent the possible links among classes denoted by annotations within a record. For instance, the subclass *Prot* $_{i_{1}}$ *_in_Org* $_{i_{1}}$ is generated to express that we deal with a protein of an organism of a certain type *Org* $_{i_{1}}$. In addition, subclasses are introduced for phenotypes, processes, cell components and molecules:

${Prot}_{i_{1}}\_Dysf\_in\_{Org}_{i_{1}}\_with\_{Phen}_{{1},\ldots,o}$ equivalentTo

*Prot* $_{i_{1}}$_*Dysf*_*in*_*Org* $_{i_{1}}$ and

‘**is part of**’ some (*Org* $_{i_{1}}$ and

(**includes** some *Phen*_1,…,*o*_))

*Prot* $_{i_{1}}$_*in*_*Org* $_{i_{1}}$_*in*_*BProc* $_{i,\ldots,_{m}}$ equivalentTo

$\phantom {\dot {i}\!}{Prot}_{i_{1}}\_in\_{Org}_{i_{1}}$ and ‘**is participant in**’ some *BProc* $_{_{1},\ldots,_{m}}$

*Prot* $_{i_{1}}$ *_in_Org* $_{i_{1}}$ *_in_CComp* $_{i_{1},\ldots,_{n}}$ equivalentTo

$\phantom {\dot {i}\!}{Prot}_{i_{1}}\_in\_{Org}_{i_{1}}$ and ‘**is included in**’ some *CComp* $_{_{1},\ldots,_{n}}$

*Prot* $_{i_{1}}$ *_in_Org* $_{i_{1}}$ *_with_Mol* $_{_{1},\ldots,_{k}}$ equivalentTo

$\phantom {\dot {i}\!}{Prot}_{i_{1}}\_in\_{Org}_{i_{1}}$ and (‘**is participant in**’ some

(*Process* and

(‘**has participant**’ some *Mol* $_{_{1},\ldots,_{k}}$)))

**Organisms**: Classes are introduced for organisms with proteins in general, and for organisms with organism-specific proteins in particular. The latter ones are also specialized by phenotypes, processes and molecules:

*Org* $_{i_{1}}$ *_with_Prot* $_{i_{1}}$ equivalentTo *Org* $_{i_{1}}$ and

‘**has part**’ some *Prot* $_{i_{1}}$

*Org* $_{i_{1}}$ *_with_Prot* $_{i_{1}}$ *_Dysf* equivalentTo *Org* $_{i_{1}}$ and

(‘**has** **part**’ some ‘*Prot* $_{i_{1}}$ *_Dysf*’)

$\phantom {\dot {i}\!}{Org}_{i_{1}}\_with\_{Phen}_{{1},\ldots,o}\_and\_{Prot}_{i_{1}}\_Dysf$ equivalentTo

*Org* $_{i_{1}}$ *_with_Prot* $_{i_{1}}$ *_Dysf* and **includes** some *P* *h* *e* *n*_1,…,*o*_

*Org* $_{i_{1}}$ *_with_Prot* $_{i_{1}}$ *_and_BProc* $\phantom {\dot {i}\!}{\!~\!}_{{1},\ldots,_{m}}$ equivalentTo

*Org* $_{i_{1}}$ and (‘**has part**’ some (*Prot* $_{i_{1}}$ and

(‘**is participant** **in**’ some $\phantom {\dot {i}\!}{BProc}_{{1},\ldots,m}$)))

$\phantom {\dot {i}\!}{Org}_{i_{1}}\_with\_{Prot}_{i_{1}}\_and\_{Mol}_{{1},\ldots,{k}}$

equivalentTo *Org* $_{i_{1}}$ and

(‘**has part**’ some *Prot* $_{i_{1}}$) and

(‘**is participant in**’ some (Process and

(‘**has participant**’ some *M* *o* *l*_1,…,*k*_)))

**Small molecules**: We introduce classes for small molecules contained in organisms, and further specify these classes by stating the type of the proteins with which these small molecules interact, i.e., with which they are related by participating in the same biological processes.

$\phantom {\dot {i}\!}{Mol}_{{1},\ldots,{k}}\_in\_{Org}_{i_{1}}$ equivalentTo *Mol*_1,…,*k*_ and

‘**is part of**’ some *Org* $_{i_{1}}$

$\phantom {\dot {i}\!}{Mol}_{{1},\ldots,{k}}\_in\_{Org}_{i_{1}}\_with\_{Prot}_{i_{1}}$

equivalentTo $\phantom {\dot {i}\!}{Mol}_{{1},\ldots,{k}}\_in\_{Org}_{i_{1}}$ and

(‘**is participant in**’ some (*Process* and

(‘**has participant**’ some *Prot* $_{i_{1}}$)))

**Processes**: Subclasses are introduced for the participating proteins which are included in a certain type of organism.

$\phantom {\dot {i}\!}{BProc}_{{1},\ldots,{m}}\_in\_{Org}_{i_{1}}\_with\_{Prot}_{i_{1}}$

equivalentTo *BProc*_1,…,*m*_ and

(‘**has participant**’ some *Prot* $_{i_{1}}$) and

(‘**is included in**’ some *Org* $_{i_{1}}$)

**Phenotypes**: Subclasses are introduced for associated dysfunctional proteins and their respective organisms.

$\phantom {\dot {i}\!}{Phen}_{{1},\ldots,{o}}\_in\_{Org}_{i_{1}}\_with\_{Prot}_{i_{1}}\_Dysf'$

equivalentTo *P* *h* *e* *n*_1,…,*o*_ and

(‘**is included in**’ some *Org* $_{i_{1}}$ *_with_Prot* $_{i_{1}}$ *_Dysf*)

The querying strategy for this representation model is to check whether specific subclasses are retrieved or not. For instance, if we want to retrieve processes with *Prot* $_{i_{1}}$_in_*Org* $_{i_{1}}$, the corresponding DL query is

*Process* and (‘**has participant**’ some *Prot* $_{i_{1}}$) and

(‘**is included in**’ some *Org* $_{i_{1}}$)

The automated reasoner delivers a list with the corresponding defined subclasses, such as:

*BProc*_1_ *_in_Org* $_{i_{1}}$ *_with_Prot* $_{i_{1}}$,

*BProc*_2_ *_in_Org* $_{i_{1}}$ *_with_Prot* $_{i_{1}}$ or

*BProc*_3_ *_in_Org* $_{i_{1}}$ *_with_Prot* $_{i_{1}}$.

A disadvantage of the SUBC interpretation is that it requires the introduction of classes that are not to be found in the ontologies used for annotation (such as GO or PRO) and that these classes are retrieved by the above query. For querying purposes, their superclasses must be identified, viz. *BProc *_1_, *BProc *_2_, and *BProc *_3_. This requires some post-processing of the results as explained below.

Thus, subclasses for all types of entities referred to in a database are created, which is on the one hand highly prolific, because every possible association of entries in table fields must be combined into a new defined class. On the other hand, the expressiveness power of the DL dialect needed is reduced to the EL++ [28], corresponding to OWL2-EL, which is known for its good scalability [28].

### Representation with dispositions (DISP)

In the representational patterns IND and SUBC, database entries were seen as observations about individuals, either represented as existing ABox entities or as specific, potentially empty, subclasses. Whereas IND makes strong existential claims, stating that the content of a field is interpreted as representing an actually existing biological individual, the ontological engagement of SUBC is more modest, as it allows empty classes (although non-denoting database entries are rather the exception than the norm). Both IND and SUBC avoid to claim any universal statement of the form “For all *A* there is some *B*” for any class A referred to by database.

In contrast, the DISP pattern goes a step further, assuming that the database content has been created to give insights into scientific regularities in the sense that all members of a class have a *disposition* to behave in a certain way, thus exhibiting a law of nature.

To ascribe a disposition for a certain process *P* to an object *m* does not imply that *m* actually and at all times participates in an instance of *P*. It implies only that the physical structure of *m* allows *m* to participate in processes of the type *P*. The proposed modelling pattern in DL is the following [29]:

*Object*_1_ and *O* *b* *j* *e* *c* *t*_2_ and …and *O* *b* *j* *e* *c* *t*_*n*_ subclassOf

‘**is bearer of**’ some (*Disposition* and

(‘**has realization**’ only *Process*_1_))

where *Object*_1_ refers to a class; and *O* *b* *j* *e* *c* *t*_2_ to *O* *b* *j* *e* *c* *t*_*n*_ refer to other classes, or to statements of the type “*ClassA* and **relation** some *ClassB*”.

The bearers of dispositions are independent continuants [19, 20]. Thus, possible bearers of dispositions, in our case organisms, proteins, small molecules and cell components.

For organisms and proteins, we create a series of general class inclusions (GCIs) in OWL, with the class of interest (e.g. *Prot* $_{i_{1}}$) intersected with the constraining conditions at the left hand side (e.g. ‘**is part of**’ some *Org* $_{i_{1}}$). Dispositions are, then, ascribed to organism-specific proteins within certain cellular components. We introduce dispositions to perform biological processes that have certain kinds of molecules as output. Here is the general pattern.

*Prot* $_{i_{1}}$ and ‘**is part of**’ some *Org* $_{i_{1}}$ subClassOf

‘**is bearer of**’ some (*Disposition* and

‘**has realization**’ only *B* *P* *r* *o* *c*_1,…,*m*_) and

‘**is bearer of**’ some (*Disposition* and

‘**has realization**’ only (*Process* and

‘**has participant**’ some *M* *o* *l*_1,…,*k*_))

In this and the next formula, the restriction

‘**is included in**’ some

(*CComp *_1_ or *CComp *_2_ or …or *CComp*_*x*_)

could be added. However, this restriction is rather weak due to the disjunction, which may leave room for several classes to be added.

As a rule, dispositions have realisation conditions. The realisation of the disposition of a protein to participate in a given biological process depends, among others, on the chemical environment within the organism and the cell component. Such dispositions are introduced for all proteins of the type *Prot* $_{i_{1}}$, under the condition that they are included in *Org* $_{i_{1}}$ as well as in one or more cellular components (*C* *C* *o* *m* *p*_1,…,*n*_). These dispositions are defined in terms of the process types $\phantom {\dot {i}\!}{BProc}_{{1},\ldots,_{m}}$ processes, or in terms of unspecified processes in which one or more small molecules ($\phantom {\dot {i}\!}{Mol}_{{1},\ldots,_{k}}$) participate.

Our interpretation of the example is that the ability to exhibit a certain pathological phenotype is attributed to organisms in virtue of having a dysfunctional protein. Again, the table does not tell us which kind of dysfunction affects which kind of process that results in which phenotype:

*Org* $_{i_{1}}$ and (**includes** some (*Prot* $_{i_{1}}$ and

(‘**is bearer of**’ some *Dysfunctional*))) subClassOf

‘**is bearer of**’ some (*Disposition* and

(‘**has realization**’ only *P* *h* *e* *n*_1,…,*o*_))

Formally, we could characterize a class of small molecules as bearing dispositions in the following way:

*Mol*_1_ or *Mol*_2_ or …or *Mol*_*k*_

subclassOf ‘**is bearer of**’ some (*Disposition* and

(‘**has realization**’ only (*Process* and

(‘**has participant**’ some *Prot* $_{i_{1}}$) and

(‘**is included in**’ some *Org* $_{i_{1}}$) and

(‘**is included in**’ some

(*CComp *_1_ or *CComp *_2_ or …or *CComp*_*n*_)))))

As we said, dispositions could theoretically also be ascribed to cell components, as these are also independent continuants. However, according to the shared background assumptions of biologists, cellular components are not participants but only the locations of the biomolecular processes under scrutiny. That an entity bears a disposition of being the arena in which a process might take place would require the extension of either the notion of disposition or the notion or participation. Therefore, we refrain from ascribing dispositions to cell components.

The use of general class inclusions (GCIs), i.e. the use of complex class expressions on the left hand side of the axiom, is a straightforward application of the above pattern. However, this strategy does not support retrieval purposes, as DL queries only retrieve simple names of classes or individuals, but not complex expressions.

### Hybrid class-level representation (HYBR)

To avoid complex class expressions on the left hand side of GCIs, a feasible approach that supports DL queries on dispositions would require equivalence axioms as the following:

*Org* $_{i_{1}}$ *_with_Prot* $_{i_{1}}$ *_Dysf* equivalentTo *Org* $_{i_{1}}$ and

(‘**has part**’ some (*Prot* $_{i_{1}}$ and

(‘**is bearer of**’ some *Dysfunctional*)))

Here, *Dysfunctional* is a class that qualifies a given *Prot* $_{i_{1}}$ as being causally related to a pathological phenotype.

The class *Org* $_{i_{1}}$ *_with_Prot* $_{i_{1}}$ *_Dysf* can then be used on the left hand side of an axiom that states the dispositions of organisms of the type *Org* $_{i_{1}}$ under the condition of having dysfunctional proteins of the type *Prot* $_{i_{1}}$. This corresponds to the modelling pattern SUBC.

In our example, this means that the SUBC model requires *n* defined classes for “organisms of the type *Org* $_{i_{1}}$ that have dysfunctional proteins of the type *Prot* $_{i_{1}}$ and which include a phenotype *P* *h* *e* *n*_1,…,*o*_”, whereas the DISP approach requires one axiom with “organisms of the type *Org* $_{i_{1}}$ that have dysfunctional proteins of the type *Prot* $_{i_{1}}$” at the left hand side, with expressions on *P* *h* *e* *n*_1,…,*o*_ at the right hand side:

*Org* $_{i_{1}}$ *_with_Prot* $_{i_{1}}$ *_Dysf* subClassOf

‘**is bearer of**’ some (*Disposition* and

(‘**has realization**’ only *P* *h* *e* *n*_1,…,*o*_))

This leads to a hybrid approach in which subclass definitions are still needed. The hybrid representation may be preferred as being more parsimonious, which however has to be traded off against the increase in DL expressiveness, viz. from OWL-EL to OWL-DL, at least when DISP (like proposed for SUBC) avoiding generation of a huge number of very specific subclasses, as in SUBC.

### Evaluating representation scenarios

We created four DL queries (Q1–Q4) (cf. Table 3) to evaluate (i) database content retrieval, using ontologies as query vocabulary; (ii) information completeness; and (iii) DL complexity and decidability. Q1 aims at retrieving biological processes in which certain proteins participate; Q2 aims at retrieving the cellular component(s) a given organism includes, together with the proteins found in them. Q3 aims at retrieving proteins recorded as participant of biological processes in a given organism. Finally, Q4 aims at retrieving organisms able to exhibit a specific phenotype.

Queries on SUBC or HYBR models require further processing, because they retrieve the subclasses introduced in the models, e.g., *P* *h* *e* *n*_1,…,*k*__*in*_*Org* $_{i_{1}}$_*with* *Prot* $_{i_{1}}$_*Dysf*, whereas the user is only interested in retrieving the classes used in the annotation, such as *P* *h* *e* *n*_1,…,*k*_ in our case.

This is easily achieved by extracting the original class names from the constructed names of each retrieved class; e.g., *P* *h* *e* *n*_1,…,*k*_ is extracted from $\phantom {\dot {i}\!}{Phen}_{{1},\ldots,{k}}\_in\_{Org}_{i_{1}}\_with\ {Prot}_{i_{1}}\_Dysf$.

Results from Q1–Q4 are displayed in Table 4. Apart from the OWL profiles required, the result shows how individuals can be retrieved with IND, and classes in two-step queries for SUBC and HYBR. DISP does not retrieve anything due to the use of GCIs without class definitions.

**Table 4:** Query results together with characteristics of the four ontology implementations (without importing BTL2)

Model	Q1	Q2	Q3	Q4	Classes	Axioms	Individuals	OWL profile
IND	**bp**1001,	**cc**1001,	p1004	–	24	207	51	OWL-DL
	**bp**2001,	**cc**2001,						
	**bp**3001	**cc**3001						
SUBC	*BProc*_1_	*CComp*_1_	*Prot*_*i*1_	–	68	149	0	OWL-EL
DISP	–	–	–	–	29	70	0	OWL-DL
HYBR	*BProc*_1_	*CComp*_1_	*Prot*_*i*1_	*Org*_*i*1_	48	129	0	OWL-DL

As expected, SUBC generates more classes and axioms than DISP and HYBR. In IND, there are more axioms than in SUBC, DISP and HYBR due to the large amount of relationships created among the individuals while an OWL model following the IND strategy may not include any class definitions. IND and SUBC were not able to retrieve Q4, which includes a disposition axiom and can be answered only by HYBR.

In the context of an integrative framework, combining “ontologised” databases and bio-ontologies, interesting variations of these competency questions can be imagined. These variations can exploit the axiomatic content of the linked ontologies, such as subclass axioms or role restrictions. Expressed in DL queries, these variations would require none or minor syntactic variations:

- In Q1, a query could target a number of biological processes by a common ancestor process, or a phase of a certain process provided by GO;
- In Q2 and Q3, the organism could be substituted by a biological taxon or other groupings of organisms, such as provided by the NCBI taxonomy or SNOMED CT (organism branch);
- In Q1 and Q3, processes could be clustered by querying for metabolite characteristics. This can be (for instance) provided by GO extensions, like the GO – ChEBI linkage.
- In Q4, phenotypes could be queried through how they are characterised, for instance by certain body locations. This can be achieved such as provided by SNOMED CT body structure and disorder.

Users should choose an interpretation approach that accounts for their respective requirements and fits to the computational resources available. With IND, the whole semantic expressivity belongs to the ontology the individuals are imported into; there is no guarantee that this ontology is expressive enough to support reasoning and querying, whereas the patterns provided by SUBC and HYBR come with axioms that fulfil this task.

Our results indicated that DISP and HYBR promise better results when reasoning over biomedical databases. However, limitations may arise for these approaches due to the nontrivial use of dispositions and scalability problems, because the reasoning complexity increases with higher expressivity. In these respects, SUBC might be the most parsimonious solution, as it may be less problematic for scaling when applying reasoning and performing queries, with the expense of simulating relations to avoid the complexity that comes with the use of dispositions.

## Discussion

Recently, ontology-aided interpretation of databases has emerged as a research topic in the biomedical domain, e.g., for disambiguating the sense of free-text keywords in query generation to access data repositories [30], or as a means to interpret proteomics data [31]. As biomedical observation databases, (e.g.) for proteomics, are still interpreted manually [7], led to the suggestion of annotation tools that support data interpretation. In these works, authors suggest a deeper use of ontologies to support interpretation, which is something that goes beyond of what is currently performed with functional annotations.

Aiming to attain this purpose, we have proposed four representation strategies: IND, SUBC, DISP and HYBR.

### Interpreting data as individuals (IND)

The representation pattern IND is completely based on single individuals (ABox entities), present in the underlying experimental assays the results of which are referred to by the database content. This approach, similarly to ontology population [32], refrains from raising any ontological claim apart from asserting the existence of individuals and relations among them. The ABox entities can then be retrieved by DL queries, but the performance problems of large ABoxes with expressive TBoxes are known [47] and may therefore hamper the theoretical issue of scalability. In addition, the assertion of existence is an estimation, because data may exhibit errors, especially when not manually curated and, e.g., extracted from literature abstracts by natural language processing.

#### IND and Ontology-based Data Access

Previously, OWL models have been created in which OWL axioms and assertions were automatically generated from database schemes [33]. These models, however, represent (first of all) data (information entities) and not the reality denoted by the data. Our approach, in contrast, aims at representing the latter, e.g, to which classes the information entities denotes and further relations among them. In addition, relations extracted from databases are semantically idiosyncratic and shallow, e.g., neglecting the complexity of the underlying reality, of which a database schema represents nothing more than a customized view.

For instance, database integration following the Ontology-Based Data Access [34] (OBDA) approach relies on a limited set of ontological relations that are provided by ontologies. In OBDA, integration relies on connecting information present in databases with ontologies, without discussing which interpretation of the data is more appropriate, i.e., whether the data refer to individuals, classes, or classes of disposition bearers (neither of which is expressed in the database nor defined in the ontology). In practice, OBDA enables the user to retrieve individuals from a database virtually, e.g., by means of an ontology used as query vocabulary and an engine to convert queries in SPARQL [35] to its respective SQL equivalent, or retrieve RDF triples such as in Bio2RDF [36] or the UniProt SPARQL Endpoint [37]. Such interpretation issues may be not so relevant for daily database usage, e.g., accessing or retrieving queries; but for biological databases, which include data from real experiments, raising them is quite relevant.

Approaches that rely on SPARQL queries, like OBDA, do not go further into how data are to be interpreted, which is crucial for the biomedical domain. E.g., queries created in SPARQL and ontologies formalized in OWL employ different semantics, e.g., of which the latter enables more complex reasoning tasks (e.g.,classification and consistency checking) than the former. Reasoning is crucial for validating content interpreted according to the semantics provided by ontologies, which frequently employ OWL.

Opposed to the stance that ontology artefacts should, first, represent purpose-oriented data structures, where different use cases might require different, partly incompatible design decisions [38], we reinforce the interoperability aspect of ontologies, which we consider to be “representational artefacts whose representational units are intended to designate classes or types in reality and to relate them to each other” [39], which also requires agreement on a set of high-level categories and relations.

#### Databases and temporal contexts

Ceusters and Smith [40] describe an approach called *Referent Tracking*, which is mainly devoted to the identification of individuals from Electronic Health Records (EHR). Referent tracking is based on the generation of triples in order to record how individuals are related to each other within a specific context. This approach is similar to our IND strategy, but equally affected by the problems of non-referring representational units [41], e.g., in case of false diagnoses or abandoned care plans.

The domain upper-level ontology BTL2 had been created with the purpose of enforcing temporal contexts for continuant individuals [15]. Whereas in EHRs time indexing is necessary to represent patients’ histories, the biological annotation case described in this paper refrains from temporal indexing, which may become relevant when further describing the annotation process itself, where temporal changes occur as data is automatically annotated and later reviewed by human curators.

### Interpreting data as subclasses (SUBC)

The inability to represent non-denoting database information was addressed by the SUBC modelling patterns which created a defined subclass for each putative referent. Our approach for this modelling is agnostic to whether such classes are instantiated or empty, as their only rationale is to act as referents of information entities in the database. Therefore, this representation can (in a way) be considered ontologically neutral in the sense that we only describe potentially instantiated classes without being committed to the actual existence of any instances. Instead, the OWL model for SUBC exemplify a way to represent discourse, regardless of whether meaningful or nonsensical. However, we have shown that an OWL-EL extract represented with SUBC successfully retrieves the desired database content.

On many occasions, researchers already use ontology terms in biological databases to express relations among classes, such as that in certain types of organisms, certain biological processes are performed by or with the aid of certain proteins. In such cases, the SUBC modelling is more natural and will reflect the observed reality.

However, one has to deal with a problem that so often appears in the area of knowledge representation, known as the frame problem. When one ascribes a certain logical property to a class, it means that all members should possess it. But in biology, there are always exceptions and variations that arguably falsify universal statements about classes. This “all-or-nothing” stance can be seen as a drawback of the SUBC approach, which has been extensively discussed. The usefulness of a SUBC approach has been proven in practice in the realms of knowledge representation applications; nevertheless, proposals to accommodate exceptions [42], modal [43], and even probabilistic, fuzzy solutions [44] have appeared both in KR and DL [45, 46].

### Interpreting data with dispositions (DISP) and the hybrid representation (HYBR)

The DISP and HYBR representation strategies, attempts to extract ontological statements in a stricter sense, i.e. accounts of scientific laws expressed by universally quantified statements about all members of a class. This is possible by introducing dispositions, e.g., by stating that all organisms with a certain dysfunctional protein are predisposed to develop certain pathological phenotypes under certain conditions only.

The DISP approach may be considered ontologically problematic, as it is quite promiscuous in ascribing dispositions on class level. What is observed in an experiment is the outcome of a particular process (which might be a collective process). From the observation of the outcome, it is inferred that particular process happened, which gives support to the assumption that the participating particulars have had the disposition to participate in such a process.

The problem lies in the extrapolation from the observation of a single case to all members of a certain class – such inductive inferences are notoriously difficult. They may be quite safe when describing the behaviour of small molecules: knowing that one particular molecule has a certain disposition, we can quite safely assume that other molecules of the same kind share this disposition, as we can think of no intrinsic property that could make a difference here. However, on the biological level, systems are much more complex. If a gene defect in a certain individual organism increases the risk for, e.g., diabetes mellitus, it does not exclude the possibility that in other organisms with the same gene defect there is no such risk. We would, that is, not be justified to ascribe an increased diabetes risk to the latter population (though we were justified to ascribe them a certain tendency to do so [19]).

There is no principled contradiction between SUBC and DISP. The fact that the class inclusion axioms proposed in DISP to introduce conditions are not suitable for DL querying, approximates the second and the third modelling approach in the sense that the latter also benefits from fully defined subclasses. Therefore, the combination of these two modelling styles (HYBR) proved to yield the best retrieval results with all four competency questions.

### General remarks

In this sense, the need for analysing and formalising the reality behind the database schemes was confirmed by our effort when creating and querying ontologically founded interpretation models. Current use of biological databases might indeed demonstrate that a flat tabular structure with the fields Protein, Organism, Process, Cellular component, Molecule and Phenotype might work for most standard queries. Its ontological interpretation under a common upper-level representation aiming at a formal description of the domain itself and not just of a specific view thereof, creates added value for more complex queries that require semantic and not only syntactic integration of biomedical ontology resources.

Entries from biomedical databases derive mostly from harvesting scientific literature or, otherwise, from the results of experiments. The veracity of these reports can be roughly assumed, but any precise representation should take into account that experimental, measurement, reporting, and curation errors might occur, so that a certain number of entries in biological databases may be false or even contradictory. This requires accounting for the underlying domain knowledge that does not surface in the database schema. Examples for these missing links are, in our examples, that the phenotypes listed in the database record are at least partly conditioned by protein dysfunctions.

We do not claim that our interpretation approach is the only possible one, or that it is exhaustive. In any case, it might be incomplete and should therefore require refinement and extension by domain experts. For example, a phenotype might not only be the result of the dysfunction of a protein, but may also be caused by the complete absence of this protein in an organism.

The real world applicability of the proposed approaches has to be assessed with large datasets in the light of computational constraints.

## Conclusion

Interpretations of biological database content tend to be ambiguous. Accordingly, we formulated the following questions:

i. How can the implicit knowledge about entities and relationships described in the structure of a biological database be represented?
ii. How can the content of databases be interpreted, i.e., which domain entities are represented by the data elements and their connections?
iii. Are structure and content of biological databases of ontological nature?
iv. If this is the case, how can they be translated into axioms or assertions in a commonly used ontology language, and which representational patterns might be considered?

Answering (i), we presented a method that formalises the implicit knowledge behind the schemas of databases like UniProt and Ensembl. In order to account for (ii), we grounded all classes in an expressive upper-level ontology. The result is (iii) a seamless representation of database structure, content and annotations as (iv) an OWL model.

Four different ontological interpretations of database content were developed and compared. The first and the second strategy represent data individuals denoting either individual processes and their participants (IND), or defined classes of such entities, using maximally expressive OWL class terms (SUBC), respectively. The third strategy (DISP) makes stronger claims by universally ascribing dispositions to some of the continuant classes involved. The fourth strategy (HYBR) combines elements from SUBC and DISP.

The usefulness of the representations was assessed by a series of competency questions formalised as DL queries, for which the hybrid representation of database referents as subclasses together with dispositions (HYBR) yielded the most convincing result when considering expressivity and reasoning. However, the SUBC may be well suited for automating interpretation, as its expressiveness scales better for reasoning tasks over a large amount of data.

Adding dispositional properties may constitute a useful add-on, although it is epistemically problematic to automate the ascription of dispositions to classes based on cursory evidence on sample individuals gathered in lab experiments.

## Additional files

Additional file 1 Sample data with records retrieved from UniProt and Ensembl. (XLSX 15.4 kb)

Additional file 2 IND representation example. (OWL 37.3 kb)

Additional file 3 SUBC representation example. (OWL 69.2 kb)

Additional file 4 HYBR representation example. (OWL 31.6 kb)

Additional file 5 DISP representation example. (OWL 14.7 kb)

## References

[1] The UniProt Consortium (2015). UniProt: a hub for protein information. Nucleic Acids Res.

[2] Natale D, Arighi CN, Blake J, Bult CJ, Christie KR, Cowart J, D’Eustachio P, Diehl AD, Drabkin HJ, Helfer O, Huang H, Masci AM, Ren J, Roberts NV, Ross K, Ruttenberg A, Shamovsky V, Smith B, Yerramalla MS, Zhang J, Aljanahi A, Çelen I, Gan C, Lv M, Schuster-Lezell E, Wu CH (2014). Protein Ontology: A controlled structured network of protein entities. Nucleic Acids Res.

[3] The Gene Ontology Consortium (2014). Gene Ontology Consortium: going forward. Nucleic Acids Res.

[4] Smith B, Ashburner M, Rosse C, Bard J, Bug W, Ceusters W, Goldberg LJ, Eilbeck K, Ireland A, Mungall CJ, Leontis N, Rocca-Serra P, Ruttenberg A, Sansone SA, Scheuermann RH, Shah N, Whetzel PL, Lewis S (2007). The OBO Foundry: coordinated evolution of ontologies to support biomedical data integration. Nat Biotechnol.

[5] Schulz S, Grewe N, Röhl J, Schober D, Boeker M, Jansen L. Guideline on Developing Good Ontologies in the Biomedical Domain with Description Logics. Technical Report December, Universität Rostock. 2012. http://purl.org/goodod/guideline.

[6] The UniProt Consortium. UniProt Core. 2017. ftp://ftp.uniprot.org/pub/databases/uniprot/current_release/rdf/core.owl. Accessed 13 Mar 2017.

[7] Laukens K, Naulaerts S, Berghe WV (2015). Bioinformatics approaches for the functional interpretation of protein lists: From ontology term enrichment to network analysis. PROTEOMICS.

[8] Baader F, Calvanese D, McGuinness DL, Nardi D, Patel-Schneider P (2007). The Description Logics Handbook: Theory, Implementation, and Applications.

[9] W, 3C. OWL 2 Web Ontology Language Document Overview. 2012. http://www.w3.org/TR/owl2-overview/.

[10] Santana F, Schober D, Medeiros Z, Freitas F, Schulz S (2011). Ontology patterns for tabular representations of biomedical knowledge on neglected tropical diseases. Bioinformatics.

[11] Hoehndorf R, Dumontier M, Gennari JH, Wimalaratne S, de Bono B, Cook DL, Gkoutos GV (2011). Integrating systems biology models and biomedical ontologies,. BMC Syst Biol.

[12] Schulz S, Boeker M, Martinez-Costa C (2017). The BioTop Family of Upper Level Ontological Resources for Biomedicine. Stud Health Technol Inform.

[13] Arp R, Smith B, Spear AD (2015). Building Ontologies with Basic Formal Ontology.

[14] Smith B, Ceusters W, Klagges B, Köhler J, Kumar A, Lomax J, Mungall C, Neuhaus F, Rector AL, Rosse C (2005). Relations in biomedical ontologies,. Genome Biol.

[15] Jansen L, Grewe N, Jansen L, Boeker M, Herre H, Loebe F (2014). Butterflies and Embryos: The Ontology of Temporally Qualified Continuants. ODLS.

[16] Glimm B, Horrocks I, Motik B, Stoilos G, Wang Z (2014). HermiT: An OWL 2 Reasoner. J Autom Reason.

[17] Horrocks I, Kutz O, Sattler U. The Even More Irresistible SROIQ In: Doherty P, Mylopoulos J, Welty CA, editors. Proc. of the 10th Int. Conf. on Principles of Knowledge Representation and Reasoning (KR-06). AAAI Press: 2006. p. 57–67.

[18] Horridge M, Patel-Schneider PF. OWL 2 Web Ontology Language: Manchester Syntax. 2009. http://www.w3.org/TR/owl2-manchester-syntax/.

[19] Jansen L (2007). Tendencies and other Realizables in Medical Information Sciences. Monist.

[20] Röhl J, Jansen L (2011). Representing dispositions. J Biomed Semant.

[21] Schulz S, Spackman K, James A, Cocos C, Boeker M (2011). Scalable representations of diseases in biomedical ontologies,. J Biomed Semant.

[22] Schulz S, Jansen L (2009). Molecular Interactions: On the Ambiguity of Ordinary Statements in Biomedical Literature. Appl Ontol.

[23] Cunningham F, Amode MR, Barrell D, Beal K, Billis K, Brent S, Carvalho-Silva D, Clapham P, Coates G, Fitzgerald S, Gil L, Giron CG, Gordon L, Hourlier T, Hunt SE, Janacek SH, Johnson N, Juettemann T, Kahari AK, Keenan S, Martin FJ, Maurel T, McLaren W, Murphy DN, Nag R, Overduin B, Parker A, Patricio M, Perry E, Pignatelli M, Riat HS, Sheppard D, Taylor K, Thormann A, Vullo A, Wilder SP, Zadissa A, Aken BL, Birney E, Harrow J, Kinsella R, Muffato M, Ruffier M, Searle SMJ, Spudich G, Trevanion SJ, Yates A, Zerbino DR, Flicek P (2014). Ensembl 2015. Nucleic Acids Res.

[24] Selhub J (1999). Homocysteine metabolism,. Annu Rev Nutr.

[25] Hoehndorf R, Dumontier M, Oellrich A, Rebholz-Schuhmann D, Schofield PN, Gkoutos GV (2011). Interoperability between biomedical ontologies through relation expansion, upper-level ontologies and automatic reasoning. PLoS One.

[26] Gangemi A, Guarino N, Masolo C, Oltramari A, Oltramari R. Understanding Top-Level Ontological Distinctions In: Gomez-Perez A, Gruninger M, Stuckenschmidt H, Uschold M, editors. Proc. of the IJCAI-01 Workshop on Ontologies and Information Sharing, vol. 47. CEUR Workshop Proceedings: 2001. p. 26–33.

[27] Schulz S, Brochhausen M, Hoehndorf R. Higgs Bosons, Mars Missions, and Unicorn Delusions: How to Deal with Terms of Dubious Reference in Scientific Ontologies, In: Bodenreider O, Martone ME, Ruttenberg A, editors. Proc. of the 2nd International Conference on Biomedical Ontology (ICBO) 2011, vol. 833. CEUR Workshop Proceedings: 2011. p. 183–9.

[28] Baader F, Brandt S, Lutz C. Pushing the EL envelope further In: Clark K, Patel-Schneider PF, editors. Proc. of the Fourth OWLED Workshop on OWL: Experiences and Directions, vol. 496. CEUR Workshop Proceedings: 2008.

[29] Schulz S, Jansen L (2013). Formal Ontologies in Biomedical Knowledge Representation. IMIA Yearbook.

[30] Bobed C, Mena E (2016). QueryGen: Semantic interpretation of keyword queries over heterogeneous information systems. Inf Sci.

[31] Carnielli CM, Winck FV, Paes Leme AF (2015). Functional annotation and biological interpretation of proteomics data. Biochim Biophys Acta.

[32] Petasis G, Karkaletsis V, Paliouras G, Krithara A, Zavitsanos E (2011). Ontology population and enrichment: State of the art. LNCS.

[33] Jain V, Prasad S (2014). Mapping Between RDBMS And Ontology: A Review. IJSTR.

[34] Poggi A, Lembo D, Calvanese D, De Giacomo G, Lenzerini M, Rosati R (2008). Linking data to ontologies. LNCS.

[35] Harris S, Seaborne A. SPARQL 1.1 Query Language. 2013. http://www.w3.org/TR/sparql11-query/.

[36] Belleau F, Nolin MA, Tourigny N, Rigault P, Morissette J (2008). Bio2RDF: Towards a mashup to build bioinformatics knowledge systems. J Biomed Inform.

[37] The UniProt Consortium. UniProt SPARQL Endpoint. 2017. http://sparql.uniprot.org/. Accessed 13 Mar 2017.

[38] Noy NF, McGuinness DL. Ontology Development 101: A Guide to Creating Your First Ontology. Stanford knowledge systems laboratory technical report KSL-01-05 and Stanford medical informatics technical report SMI-2001-0880. Stanford, CA; 2001.

[39] Schulz S, Johansson I (2007). Continua in Biological Systems. Monist.

[40] Ceusters W, Smith B (2006). Strategies for referent tracking in electronic health records. J Biomed Inform.

[41] Smith B, Ceusters W. Aboutness: Towards foundations for the information artifact ontology In: Couto FM, Hastings J, editors. Proc. of the International Conference on Biomedical Ontology (ICBO) 2015, vol. 1515. CEUR Workshop Proceedings: 2015. p. 1–5.

[42] McCarthy J, Hayes PJ (1969). Some philosophical problems from the standpoint of artificial intelligence. Mach Intell.

[43] Kripke SA (1963). Semantical Considerations on Modal Logic. Acta Philosophica Fennica.

[44] Zadeh LA (1965). Fuzzy sets. Inform Control.

[45] Niepert M, Noessner J, Stuckenschmidt H. Log-Linear Description Logics In: Walsh T, editor. Proc. of the Twenty-Second International Joint Conference on Artificial Intelligence. AAAI Press: 2011. p. 2153–158. doi:10.5591/978-1-57735-516-8/IJCAI11-359.

[46] Casini G, Meyer T, Moodley K, Varzinczak I. Proceedings of the 26th International Workshop on Description Logics In: Eiter T, Glimm B, Kazakov Y, Krötzsch M, editors.. CEUR Workshop Proceedings: 2013. p. 364–76.

[47] Hustadt U, Motik B, Sattler U. Data Complexity of Reasoning in Very Expressive Description Logics. In: Proceedings of the 19th International Joint Conference on Artificial Intelligence. IJCAI: 2005. p. 460–465.

